# The role of brain oscillations in post-stroke motor recovery: An overview

**DOI:** 10.3389/fnsys.2022.947421

**Published:** 2022-07-29

**Authors:** Giulia Leonardi, Rosella Ciurleo, Francesca Cucinotta, Bartolo Fonti, Daniele Borzelli, Lara Costa, Adriana Tisano, Simona Portaro, Angelo Alito

**Affiliations:** ^1^Department of Physical and Rehabilitation Medicine and Sports Medicine, Policlinico “G. Martino,” Messina, Italy; ^2^IRCCS Centro Neurolesi Bonino-Pulejo, Messina, Italy; ^3^Department of Biomedical, Dental Sciences and Morphological and Functional Images, University of Messina, Messina, Italy; ^4^Department of Clinical and Experimental Medicine, University of Messina, Messina, Italy

**Keywords:** brain oscillations, stroke disability, neuromodulation, rehabilitation, non-invasive brain stimulation (NIBS)

## Abstract

Stroke is the second cause of disability and death worldwide, highly impacting patient’s quality of life. Several changes in brain architecture and function led by stroke can be disclosed by neurophysiological techniques. Specifically, electroencephalogram (EEG) can disclose brain oscillatory rhythms, which can be considered as a possible outcome measure for stroke recovery, and potentially shaped by neuromodulation techniques. We performed a review of randomized controlled trials on the role of brain oscillations in patients with post-stroke searching the following databases: Pubmed, Scopus, and the Web of Science, from 2012 to 2022. Thirteen studies involving 346 patients in total were included. Patients in the control groups received various treatments (sham or different stimulation modalities) in different post-stroke phases. This review describes the state of the art in the existing randomized controlled trials evaluating post-stroke motor function recovery after conventional rehabilitation treatment associated with neuromodulation techniques. Moreover, the role of brain pattern rhythms to modulate cortical excitability has been analyzed. To date, neuromodulation approaches could be considered a valid tool to improve stroke rehabilitation outcomes, despite more high-quality, and homogeneous randomized clinical trials are needed to determine to which extent motor functional impairment after stroke can be improved by neuromodulation approaches and which one could provide better functional outcomes. However, the high reproducibility of brain oscillatory rhythms could be considered a promising predictive outcome measure applicable to evaluate patients with stroke recovery after rehabilitation.

## Introduction

A stroke is defined as a sudden onset of signs and symptoms related to focal or global cerebral deficits of brain function, lasting more than 24 h, not attributable to any apparent cause other than cerebral vasculopathy (Sacco et al., 2013). Six months post-stroke, nearly 50% of survivors have some residual motor deficits (Benjamin et al., 2017). Advances in acute stroke therapeutic management (intravenous thrombolysis, mechanical thrombectomy) have improved the prevention possibilities of long-term disability (Tong et al., 2012). Being the second cause of disability and death worldwide (GBD 2016 Stroke Collaborators, 2019), stroke has high relevance to a patient’s quality of life and significant impact on health care costs. Functional impairment, resulting in poor performance in activities of daily living, is common (Benjamin et al., 2017). Environmental conditions are required for post-stroke motor recovery (Power et al., 2011; Wenger et al., 2017). Internal processes combinations such as functional undamaged neural structures recovery and/or brain network remapping could promote impaired functions spontaneous restoration (Gazzaniga, 2005). The phenomenon behind these recovery processes is lifetime—continuous motor system neuroplasticity (Power et al., 2011; Remsik et al., 2016). Traditional rehabilitation techniques enhance motor function recovery (Kollen et al., 2006; Fleet et al., 2014; Laver et al., 2015) leveraging this motor learning circuitry, thus improving patient outcomes (Thakor, 2013). The relationship between brain activity and movements is important for motor learning, thus integrating motor system modulation and rehabilitation techniques in treatment settings could aid stroke recovery (Pfurtscheller et al., 2005; Felton et al., 2007; Schalk et al., 2008). Several neurological disorders (i.e., stroke) are associated with altered electroencephalogram (EEG) brain rhythms, which sustain motor, cognitive, and perceptive functions (Muralidharan et al., 2011; Ortner et al., 2012). EEG signal oscillations detectable in sensorimotor areas, especially in the mu (8–13 Hz) and beta (13–30 Hz) bands, present characteristic modulation during motor tasks. Interestingly, alpha and beta rhythms modulations caused by sensory stimulation, a motor act or motor imagery, are correlated with a decrease or increase in the underlying neuronal population’s synchrony (McFarland et al., 2000; Pfurtscheller et al., 2006; Nicolas-Alonso and Gomez-Gil, 2012). Modulations of sensorimotor rhythms resulting from sensory stimulation, motor act, or its imagination can be of two types, namely, event-related desynchronization (ERD) and event-related synchronization (ERS) of mu and beta rhythms (Jeannerod, 1995; Pfurtscheller and Neuper, 2001**). Specifically, ERDs consist of a decrease in the amplitude of rhythms, while ERS is an increase in the amplitude of rhythms (Felton et al., 2007).** Alpha (mu) and beta oscillations can be used as control rhythms for a “brain–computer interface” (BCI) system (Schalk et al., 2004). BCI systems can transform brain activity into control signals for external devices (Schalk et al., 2004; McFarland and Wolpaw, 2011; Lee et al., 2020), and can be used for tasks that require users to activate or deactivate specific brain regions (Rathee et al., 2019). Therefore, non-invasive BCI systems can facilitate recovery in patients with chronic post-stroke by linking brain activity with distal motor effectors in the peripheral nervous system (Song et al., 2014). Feedback-regulated motor imagination could be used to improve functional recovery, enhancing antagonistic ERD/ERS patterns, and, consequently, supporting stroke-affected hemisphere activation and contralateral unaffected hemisphere inhibition (Pfurtscheller and Neuper, 2006). Therefore, in the BCI system, brain activity can be transformed into control signals for external devices including “functional electrical stimulation” (FES) (McFarland and Wolpaw, 2011). Thus, non-invasive EEG-BCI-FES systems may facilitate recovery in patients with chronic post-stroke by linking brain activity with distal motor peripheral nervous system effectors and may be used as biomarkers to predict rehabilitation outcomes (Song et al., 2014, 2015).

To modulate and explore brain function, non-invasive brain stimulation (NIBS) could be applied. To date, there are different NIBS protocols with therapeutic applications, reflecting synaptic mechanisms of long-term potentiation (LTP) or long-term depression (LTD), even in stroke rehabilitation (Terranova et al., 2019). The NIBS after effects are short lasting (∼30–120 min) in humans (Abraham and Williams, 2003), but other mechanisms are also involved [i.e., post-tetanic potentiation (PSP) and short-term potentiation (STP)] (Ugawa, 2012). The most applied NIBS are transcranial magnetic stimulation (TMS), transcranial direct current stimulation (tDCS), transcranial alternating current stimulation (tACS), and transcranial random noise stimulation (tRNS) (Paulus, 2011; Terranova et al., 2019). TMS motor-evoked potentials are obtained from the contralateral muscles of the stimulated hemisphere (Barker et al., 1985). TMS can modulate cortical excitability in different ways: (i) Inducing electrical field causing local effects immediately under the coil and/or remote effects (i.e., excitatory and inhibitory effects) (Rothwell et al., 1999) and (ii) applying a transient weak current to the brain through a pair of saline-sponged electrodes (Nitsche et al., 2008) and changing the polarity of the current. Repetitive transcranial magnetic stimulation (rTMS) produces long-term changes, reducing cortical excitability at low frequency (≤ 1 Hz), and boosting it up at high frequency (≥ 5 Hz) (Maeda et al., 2000; Siebner and Rothwell, 2003; Quartarone et al., 2005). However, it has been shown that continuous 5 Hz rTMS decreases instead of increasing corticospinal excitability (Rothkegel et al., 2010). When rTMS is administered in a complex burst pattern, i.e., theta burst stimulation, it produces more reliable effects than conventional rTMS (Huang et al., 2005; Hamada et al., 2008; Suppa et al., 2016). Another rTMS approach, namely, theta burst stimulation (TBS) (intermittent or continuous), uses 5 Hz short bursts at a repetitive high frequency mimicking the brain’s natural firing patterns (Oberman et al., 2011; Hoy et al., 2016). Compared to rTMS, intermittent TBS (iTBS) may be applied to induce greater and longer-lasting motor cortical effects on cortical excitability (Huang et al., 2005; Di Lazzaro et al., 2008). It is applied using biphasic stimulus pulses that induce an initial posterior-anterior current through M1 (Huang et al., 2005). The use of short 5-Hz high-frequency repetitive bursts that mimic the brain’s natural firing patterns would result in greater neuromodulatory potential than the standard approach. Thus, the effects on the functional brain network of patients with stroke would be greater and longer lasting in regions remote from the stimulated site (Oberman et al., 2011; Hoy et al., 2016; Suppa et al., 2016). Continuous TBS (cTBS) decreases cortical excitability, while intermittent TBS has a booster-up effect (Hamada et al., 2008). However, tDCS is mainly applied in clinical practice, while tACS and tRNS are more used in a research context (Paulus, 2011). Anodal tDCS modulates the cortical excitability of depolarizing neurons, whereas cathodal tDCS reduces the excitability of hyperpolarizing neurons (Antal et al., 2004). In 1–2 mA tDCS, electrical current is delivered over the skull through sponge electrodes, changing neurons firing frequency (Paulus, 2011); anodal stimulation induces cortical facilitation, whereas cathodal stimulation has an opposite effect (Paulus, 2011). However, despite TMS and tDCS having different mechanisms of action (acting TMS as neurostimulator and tDCS as neuromodulator), they both induce cortical excitability long-term after effects, which engage neural plasticity mechanisms (Fregni et al., 2005; Khedr et al., 2010). Transcranial alternating current stimulation (tACS) is a variant of TMS at a predetermined frequency (Alekseichuk et al., 2016). Transcranial random noise stimulation (tRNS) is another NIBS technique using a low-intensity biphasic randomly alternating current at a variable frequency (Fertonani et al., 2011). While researchers are still debating over the functional meaning of these synchronization and de-synchronization patterns of rhythmic activity, practical applications based on the accumulated knowledge are already emerging. On such a basis, this review aims to evaluate the role of brain oscillatory activity on motor function recovery in patients with post-stroke undergoing conventional rehabilitation treatment integrated with different NIBS.

## Search strategy and selection criteria

A computerized literature search was performed in Pubmed, Scopus, and the Web of Science from 2012 to 2022, using the following string: “brain rhythms” and “stroke” and “EEG.” The screening process and analysis were conducted separately by 3 independent observers.

First, the articles were screened by title and abstract. The following inclusion criteria for relevant articles were used during the screening: (1) Randomized controlled trials (RCTs), (2) English language, (3) published in indexed journals over the last 10 years (2012–2022), (4) including only adult human (>18 years), and (5) dealing with brain rhythms and their analysis and applications in stroke rehabilitation. The exclusion criteria were non-English articles, reviews, non-randomized controlled studies, and trials on other nervous system diseases different from a stroke.

In the second step, the full texts of the selected articles were screened, with further exclusions according to the previously described criteria and focusing our attention on motor functions.

**FIGURE 1:**
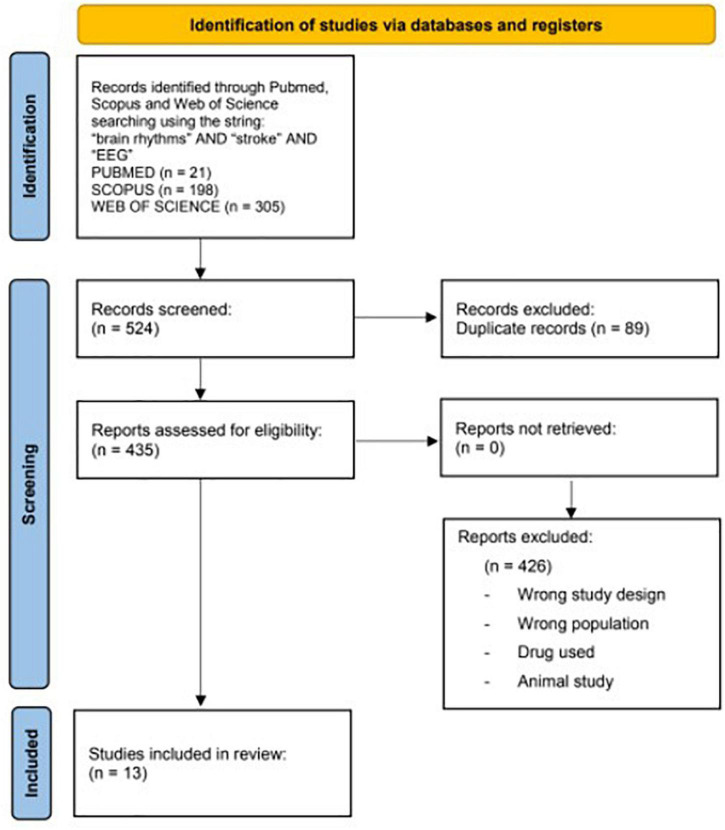
Preferred reporting items flowchart resuming the paper’s selection process.

A flowchart is given in Figure 1.

The following data were retrieved: (1) treatment groups, (2) sample size and patients’ features, (3) time since stroke, (4) therapeutic protocols, (5) outcome measures, (6) time points of follow-up evaluations, and (7) summary of clinical results.

## Intervention protocols

We analyzed the RCT discussing rehabilitative interventions on motor impairment after stroke and the applied innovative NIBS and BCI systems. We grouped the studies considering the impaired involved limb/site and the applied techniques as follows:

a) Upper limb involvement applying theta burst stimulation (TBS) or repetitive transcranial magnetic stimulation rTMS or tDCS.

Four out of thirteen articles investigated upper limb functions through transcranial stimulation (Nicolo et al., 2018; Ding et al., 2021; Dionísio et al., 2021; Kuzu et al., 2021).

Dionísio et al. (2021) compared cTBS vs. placebo stimulation, finding that contralateral inhibitory stimulation led to a significant excitatory impact on the cortical oscillatory beta band patterns of the contralateral affected hemisphere. In Ding’s (2021) study, the authors found that iTBS modulates brain network functioning in stroke survivors, normalizing brain connections, re-gaining the natural oscillation frequency, and promoting motor function recovery. Kuzu et al. (2021) showed improvements in upper extremity motor function and activities of daily living by combining physical therapy and cTBS or rTMS. However, these treatments showed limited evidence of improving spasticity (Kuzu et al., 2021). Nicolo et al. (2018) demonstrated that stimulation treatments combined with physical therapy did not enhance clinical motor gains in a heterogeneous sample of patients with sub-acute stroke.

Other studies have shown that tDCS enhanced perilesional beta-band oscillation coherence compared with cTBS and the sham groups (Carmichael, 2006; Murphy and Corbett, 2009). Moreover, tDCS produces and modulates the ongoing synaptic activity during motor activation determining a weak polarization of large assemblies of neurons (Carmichael, 2006; Murphy and Corbett, 2009). The modulation of inter-hemispheric driving and peri-lesional beta-band connectivity was related to functional recovery across all the patients (Carmichael, 2006; Murphy and Corbett, 2009). One of the potential mechanisms explaining these results could lie in the functional connectivity that increases with adaptive cortical plasticity induction caused by tDCS (Carmichael, 2006; Murphy and Corbett, 2009).

b) Upper limb involvement applying different kinds of stimulation.

Six out of thirteen articles investigated upper limb functions exploring mirror therapy and BCI (Bae et al., 2012; Ramos-Murguialday et al., 2013; Remsik et al., 2019; Lee et al., 2020; Ray et al., 2020; Sebastián-Romagosa et al., 2020). Some authors have shown that mirror therapy and its effect on mu-rhythm suppression positively influence brain activity (Bae et al., 2012). Other authors investigated the action observation training (AOT) effect associated with EEG-based BCI-controlled FES system on motor recovery of the upper extremity and cortical activation in patients with stroke (Remsik et al., 2019; Lee et al., 2020), showing that the AOT plus BCI-FES group had an increase in alpha and beta waves concentration, with improved functional outcome scores (Remsik et al., 2019; Lee et al., 2020), as previously reported (Prasad et al., 2010; McCrimmon et al., 2014; Chung et al., 2015). Other authors supported the importance of BCI devices to improve upper extremity functions in stroke survivors (Remsik et al., 2019). Intervention corresponds with greater mu-rhythm de-synchronization in the ipsilesional hemisphere during the impaired hand attempted movements (Remsik et al., 2019).

Different authors explored the efficacy of daily body mass index (BMI) in stroke survivors (Ramos-Murguialday et al., 2013; Ray et al., 2020).

Ray et al. (2020) evaluated the relationship between oscillatory sensorimotor brain activity and motor recovery in chronic stroke, identifying a correlation between alpha de-synchronization during the rehabilitative intervention and clinical improvement. These results showed that inter-hemispheric balance plays an important role in motor recovery using BMI associated with physiotherapy, which exerts an add-on effect on hand motor function recovery (Ramos-Murguialday et al., 2013).

Other authors investigated the correlation between EEG parameters, such as the “Brain Symmetry Index” (BSI) and the laterality coefficient (LC) (Sebastián-Romagosa et al., 2020). The authors calculated LC values in two frequency bands, namely, 8–13 Hz (α band, mu frequency rhythm) and 13–30 Hz (β band), disclosing the most relevant results in the alpha band (Sebastián-Romagosa et al., 2020). Specifically, the LC values calculated during M1 tasks with the healthy hand (LCh) were significant compared to those of the paretic hand MI tasks (LCp). These results showed that the LCh in the alpha band presented numerous significant correlations with functional scales, whereas the correlation between the LCp and the functional scales is less common (Sebastián-Romagosa et al., 2020). This could occur because the affected hemisphere does not present a normal activation pattern, but the healthy hemisphere maintains the normal patterns of de-synchronization during the ipsilateral motor movements. The ERD/ERS patterns observed in the healthy hemisphere should be more stable than those in the affected brain side (Sebastián-Romagosa et al., 2020).

c) Lower limb involvement applying iTBS

Ding et al. (2022) first reported that iTBS increase the natural frequency in the ipsilesional motor cortex and is useful in upper limb stroke rehabilitation. Unfortunately, such improvement has not been proven for the lower limbs (Lin et al., 2019).

d) Upper and lower limbs involvement applying tDCS

Kasashima et al. (2012) demonstrated that, in severe stroke hemiparesis, anodal tDCS could increase ERD in the mu-band, inducing long-term after-effects on cortical excitability through neural plasticity mechanisms (Fregni et al., 2005; Khedr et al., 2010). Such an effect makes tDCS a possible conditioning tool for BCI (Kasashima et al., 2012). Specifically, in the affected hemisphere, mu ERD increased after anodal tDCS during motor imagery, in both the stroke and healthy participants, possibly because of a decreased synchrony of the underlying neuronal population (Kasashima et al., 2012). Therefore, modulating mu ERD in BCI treatment could increase cortical excitability, normalizing stroke EEG-ERD values (Kasashima et al., 2012).

e) Upper and lower limbs involvement applying BCI

Tsuchimoto et al. (2019) analyzed a motor image-guided robotic approach in post-stroke hemiplegia using an EEG-based neurofeedback system. Specifically, the alpha band reflected the prominent component of activity in the sensory cortex and the beta band maintained activity in the motor cortex (Tsuchimoto et al., 2019). Moreover, the authors found that the integration of EEG and sensorimotor feedback led to increased co-activation of the sensory and motor cortices during the neurofeedback intervention (Tsuchimoto et al., 2019). On such a basis, alpha- and beta-band EEG may be used as physiological biomarkers of motor learning in stroke recovery (Tsuchimoto et al., 2019).

## Discussion

After a stroke, brain oscillatory changes are likely due to the activation of inflammatory pathways and increased oxidative stress (Moskowitz et al., 2010), leading to motor, speech, and cognitive impairments (Sun et al., 2014; Hatem et al., 2016). Neuroplasticity and exercise are used to promote recovery in stroke survivors (Alia et al., 2017; Caglayan et al., 2019; Inoue et al., 2020). Thus, NIBS may modulate brain rhythms in chronic stroke. Specifically, brain oscillations are rhythmic patterns, occurring at different frequencies (i.e., delta 1–3 Hz, theta 3–7 Hz, alpha 8–12 Hz, beta 13–25 Hz, and gamma 25–100 Hz), generated by the synchronized interaction of neuronal firing (Colgin, 2016). Multiple studies found that neural oscillations after stroke influence recovery outcomes (Rabiller et al., 2015). Specifically:

a) Just after stroke:
- Alpha oscillations are lower in frequency and more synchronized (Petrovic et al., 2017).
- Beta oscillatory power is increased in both the hemispheres (Assenza et al., 2009).
- Gamma oscillations are disrupted (Buzsáki and Wang, 2012).
b) In chronic stroke:
- Alpha oscillation de-synchronization is associated with improved motor outcomes (Westlake et al., 2012; Ray et al., 2020).
- Increased beta coherence between the motor cortex and other regions in the acute phase is associated to improve functional outcomes 3 months after stroke (Nicolo et al., 2018). Higher beta power in the affected hemisphere is associated to improve motor function, whereas in the unaffected hemisphere, it correlates with worse clinical outcomes (Thibaut et al., 2017; Espenhahn et al., 2020). Thus, in stroke recovery, changes in beta oscillations across the brain may be not congruent.
- An increase in gamma power in the affected hemisphere is a good recovery target associated with improved clinical outcomes (Tecchio et al., 2007).

To date, neuromodulation approaches, i.e., FES-NIBS, are valid tools to improve stroke rehabilitation outcomes. In addition, BCI systems could support motor function enhancement by providing visual/somatosensory feedback (Pillette et al., 2020), whereas EEG could provide real-time brain rhythms feedback (Enriquez-Geppert et al., 2017). The high reproducibility of ERD/ERS could be considered a promising predictive outcome measure for evaluating patients with stroke recovery after rehabilitation.

The core articles included in this review are shown in Table 1. Specifically, it has been shown that the above-reported techniques are more effective in the chronic stroke phase, whereas in the acute/post-acute phase, their effectiveness should be better explored. However, these data need to be cautiously taken because of several study limitations. First, this review was conducted as a broad overview of a topic-related research area. Moreover, the search method depends on a non-predefined protocol, which may involve subjective selection bias. Many of these studies have been plagued by methodological problems (i.e., the heterogeneity of the population, the rehabilitation sessions and duration, the ischemic areas involved, timing from the acute event, and the different EEG-BCI-FES-NIBS applied). The Inclusion criteria of studies for review also rely on researchers’ experiences. Finally, the selected studies reported different functional outcomes and the scarcity of the data prevented performing systematic review. However, devising novel clinical trials, which consider such limitations, may be helpful to define proper treatment settings, integrating motor system modulation and rehabilitation techniques, to shed new light on the role of brain oscillations in patients with post-stroke rehabilitation.

**TABLE 1:** Synopsis of all the RCTs included in the review.

References	Study design	Stroke phase	Patients features	Therapeutic protocol	Score baseline	Follow-up	Results	Overall performance
Bae et al. (2012)	RCT (mirror therapy vs. sham therapy)	> 6 Months	20 (10 vs. 10) **Sex:** M: 6 F: 4 vs. M: 7 F: 3 **Age:** 55.2 ± 8.5 vs. 52.6 ± 11.2	**EG:** mirror therapy for 30 min each time, 5 times per week, for 4 weeks. **CG:** sham mirror therapy	EEG, MFT, MMSE, MAS, Brunnstrom stage of hand	Prior to and after the intervention	Mirror therapy had an effect on mu rhythm suppression, improving brain activities and positively influencing motor function recovery. Therefore, it is considered to be useful as part of a rehabilitation program for subacute stroke patients.	**Mirror therapy**+
Ding et al. (2021)	Single-blind RCT (Active Intermittent theta burst stimulation vs. Sham iTBS)	> 18 Months	30 (15 vs. 15) **Sex:** M: 12 F: 3 vs. M: 9 F: 6 **Age:** 65.1 ± 11.9 vs. 61.1 ± 12.1	**EG:** Intermittent theta burst stimulation consisted of bursts containing 3 pulses at 50 Hz repeated at 5 Hz was applied over the M1 in the ipsilesional hemisphere **CG:** sham intermittent theta burst	FMA, ARAT	Resting-state EEG was recorded at baseline and immediately after iTBS	iTBS modulates brain network functioning in stroke survivors. Acute increase in interhemispheric functional connectivity and global efficiency after iTBS suggest that iTBS has the potential to normalize brain network functioning following stroke, which can be utilized in stroke rehabilitation.	**iTBS**+
Dionísio et al. (2021)	Single-blind RCT (continuous theta burst stimulation (cTBS) vs. placebo stimulation)	7 ± 3 Days	10 (5 vs. 5) **Sex:** M: 4 F: 1 vs. M: 2 F: 3 **Age:** 70.20 ± 8.701 vs. 64.00 ± 17.564	**EG:** Both single-pulse and continuous theta burst were administered for each hemisphere. The intensity which generated MEPs ranging from 0.5 to 1 mV and gave 20 single pulses at 100% of the rest intensity determined for the respective hemisphere **CG:** performed sham stimulation by reducing the intensity to zero level stimulation and using a sham noise generator.	Wolf motor function test, EEG, EMG	Before (T0), after stimulation (T1), and at 3-months’ follow-up (T2)	Excitatory response (increase in event-related desynchronization) in the sensorimotor cortical areas of the affected hemisphere, after stimulation. This contralateral inhibitory stimulation paradigm changes neurophysiology, leading to a significant excitatory impact on the cortical oscillatory patterns of the contralateral hemisphere.	**cTBS**+
Kasashima et al. (2012)	RCT (anodal tDCS vs. sham tDCS)	> 6 Months	13 (6 vs. 7) **Sex:** NA **Age:** mean age, 56.8 ± 9.5 years	**EG:** anodal tDCS over the motor area of the affected hemisphere. The order of the stimulations was randomized and the interval between the stimulation was more than 2 days. One ERD assessment session consisted of 20 trials. One trial consisted of an 8-s period of relaxation, a 2-s period of ready state, and a 5-s period of imagery. Before and after tDCS or sham stimulation, 3 sessions were conducted with approximately 5 min of rest between each session **CG:** sham tDCS	Fugl-Meyer assessment upper extremity motor score, Ashworth scale for finger flexors	Before and after anodal tDCS	Anodal tDCS can increase mu ERD in patients with hemiparetic stroke, indicating that anodal tDCS could be used as a conditioning tool for BCI in stroke patients.	**Anodal tDCS**+
Kuzu et al. (2021)	Double-blind RCT (rTMS vs. cTBS vs. sham cTBS)	> 6 Months	20 (7 vs. 7 vs. 6) **Sex:** M: 4 F: 3 vs. M: 6 F: 1 vs. M: 2 F: 4 **Age:** 56.3 ± 11.5 vs. 61.3 ± 9.8 vs. 65 ± 4.6	**EG:** 10 sessions of 20 min rTMS non-lesional hemispheric upper extremity motor area (M1) **EG:** 10 sessions of non-lesional hemispheric upper extremity motor area (M1)cTBS for 40 s **CG:** Sham cTBS PT	MAS, UE-FM, FIM, MAL-28, Brunnstrom- upper extremity brunnstrom- hand	Pre-treatment, post-treatment and at 4 weeks	Real cTBS or real rTMS combined with PT provided improvement on upper extremity motor functions and daily living activities in chronic ischemic stroke patients, but improvement in spasticity was limited	cTBS + rTMS +
Lee et al. (2020)	Single-blind RCT (AOT plus BCI-FES + conventional physical therapy vs. FES treatment and conventional physical therapy)	Within 12 months post-stroke	26 (13 vs. 13) **Sex:** M: 4 F: 9 M: 6 F: 7 **Age:** 55.15 (11.57) vs. 58.30 (9.19)	**EG:** 30 min of AOT plus BCI- FES training on the upper extremity, 20 sessions 5 times per week during the 4-week intervention period **CG:** FES treatment + conventional physical therapy	FMA-UE, WMFT, motor activity log (MAL) and modified barthel index (MBI).	Pre- prior to randomization and post-within a week after the last training session.	After intervention, there were significant differences between two groups in FMA-UE, WMFT, MAL and MBI and the results of EEG including alpha power, beta power, concentration and activation. AOT plus BCI-FES can enhance motor function of upper extremity and cortical activation in patients with stroke.	**AOT plus BCI–FES**+
Lin et al. (2019)	Single-blind RCT (TBS vs. Sham)	> 6 Months	20 (10 vs. 10) **Sex:** M: 9 F: 1 vs. M: 8 F: 2 **Age:** 60.8 ± 8.1 vs. 61.1 ± 9.7	**EG:** 10 sessions 2/week for 5 weeks of brief train of basic theta bursts (5 Hz) lasting for 2 s (10 bursts, each containing three pulses of 35 Hz) and administered every 10 s for a total of 40 trains (a total of 1,200 pulses; iTBS1200) **CG:** Sham iTBS + PT	NIHSS, BRS, mRS, FMA,	Before and after iTBS	Within-group differences were significant in the Berg balance scale for both groups, in the Fugl-Meyer assessment and overall stability index of Biodex balance system of iTBS group. No significant between-group differences were found	iTBS –
Nicolo et al. (2018)	Double- blind RCT (neuronavigated cTBS vs. cathodal tDCS vs. sham TMS/sham tDCS)	≥ 10 Weeks	41 (14 vs. 14 vs. 13) **Sex:** M:7 F:7 vs. M: 8 F:6 vs. M:8 F:5 **Age:** 62.4 ± 12.3 vs. 68.5 ± 10, 8 vs. 64.3 ± 17.1	**EG:** 9 sessions, over 3 weeks of NIBS cTBS + 30 min of PT **EG:** 9 sessions, over 3 weeks of NIBS cathodal tDCS + 30 min of PT **CG:** sham stimulation over the contralesional primary motor cortex.	UE-FMA, BBT, NHPT	Two pre-intervention baseline assessments separated by 1 week (T1 and T2), post-intervention assessments after (T3) and 30-days after stimulation treatment	Neither stimulation treatment enhanced clinical motor gains. The inhibition of the contralesional primary motor cortex or the reduction of interhemispheric interactions was not clinically useful in a heterogeneous group of subacute stroke subjects. An early modulation of perilesional oscillation coherence seems to be a more promising strategy for brain stimulation interventions.	**tDCS =**
Ramos-Murguialday et al. (2013)	RCT (BMI vs. sham BMI)	≥ 10 months	30 (16 vs. 14) **Age:** 49.3 ± 12.5 vs. 50.3 ± 12.2 **Sex:** M:9 F:7 vs. M:9 F:5	**EG:** Brain activity moved the orthoses **CG:** Random orthoses movement not linked to the control of brain oscillatory activity + PT	FMA, Ashworth Scale	8 weeks and 2 days before treatment, after 8 weeks treatments	Addition of BMI training can be used to induce functional improvements in motor function in chronic stroke patients	**BMI**+
Ray et al. (2020)	Double blind RCT (combined brain–machine interfaces and physiotherapy vs. BMI independent of brain activity)	> 8 Months	30 (16 vs. 14) **Sex:** M: 18 F: 12 **Age:** 49.8 ± 12.4	Each subject performed 17 ± 1.8 sessions of BMI-training within a period of up to 6 weeks + physiotherapy after the BMI training	FMA	At the post test and two tests prior to the intervention.	Initial alpha desynchronization might be key for stratification of patients undergoing BMI interventions and that its interhemispheric balance plays an important role in motor recovery.	**BMI**+
Remsik et al. (2019)	RCT (EEG-BCI-FES intervention vs. sham BCI)	Varied times since stroke	21 **Age:** mean age 61.6 ± 15.3 years **Sex:** M: 9 F: 12	The number of EEG-BCI-FES intervention sessions varied across subjects with a mean of 13.8 ± 1.3. Each session consisted of multiple runs of the “Cursor Task” (mean of 31.3 ± 10.5 runs per session), about 1/3rd of which included only visual feedback, and roughly 2/3 of which were comprised of BCI facilitated functional electric stimulation of the impaired hand and lingual electrotactile stimulation through a tongue display unit	ARAT, SIS, NIHSS, Barthel scale, grip strength, 9-HPT	At baseline, mid-therapy and at completion of therapy	Intervention corresponds with greater desynchronization of Mu rhythm in the ipsilesional hemisphere during attempted movements of the impaired hand and this change is related to changes in behavior as a result of the intervention.	**BCI**+
Sebastián-Romagosa et al. (2020)	RCT (BCI Cortical group vs. BCI Subcortical group vs. BCI Cortical + Subcortical group vs. rEEG)	at least 4 days before the first assessment	68 (5 vs. 17 vs. 12 vs. 32) **Age:** 57.6 ± 27.3 vs. 66.4 ± 12.7 vs. 67.0 ± 09.4 vs. 42.3 ± 15.4 **Sex:** M: 4 F: 1 M: 9 F: 8 M: 9 F: 3 M: 13 F: 19	136 Assessment sessions were performed in total (4 per patient), 4 assessment sessions (2 sessions per week) and 25 therapy sessions	FMA, Barthel index, FTRS_h, Modified Ashworth Scale, 9HPT, 1TPDT_h	Just after the last session, 1 month after the last session	Significant differences in the BSI (Brain symmetry index) between the healthy group and Subcortical group and also between the healthy and Cortical + Subcortical group. No significant differences were found between the healthy group and the Cortical group. The quantitative EEG tools used here may help support our understanding of stroke and how the brain changes during rehabilitation therapy.	**BCI**+
Tsuchimoto et al. (2019)	Double blind RCT (EEG-SMR- based neurofeedback intervention vs. sham EEG-SMR)	3–234 Months (median 6.8)	17 **Age:** 58 ± 10 y **Sex:** M: 14 M: 3	The patients were asked to imagine extension of the affected finger without actual execution during 5 s after voluntary relaxation for 5 s; thus, each trial lasted 10 s, and the decoding session was conducted without feedback for 15 trials.	FMA, SIAS	One intervention on a given day and the other intervention 1 or 2 weeks later	Although the neurofeedback intervention delivered fewer total sensorimotor stimulations compared to the sham-control, rsfcMRI in the ipsilesional sensorimotor cortices was increased during the neurofeedback intervention compared to the sham-control. Higher coactivation of the sensory and motor cortices during neurofeedback intervention enhanced rsfcMRI in the ipsilesional sensorimotor cortices.	**EEG-SMR-based neurofeedback**+

AOT, action observation training; ARAT, action research arm test; BBT, Box and Block test; BCI, brain-computer interface; BRS, Brunnstrom Stage; EEG, electroencephalography; EEG-SMR, electroencephalography signal of sensorimotor rhythm; ERD; EEG event-related desynchronization; ERS; EEG event-related desynchronization; FES, functional electronic stimulation systems, FIM, Functional Independence Measure; FMA, Fugl-Meyer motor function assessment; FTRS_h, Fahn Tremor Rating Scale for the healthy hand; rsfcMRI, functional magnetic resonance imaging; MAL-28, Motor Activity Log-28; MAS, Modified Ashworth Scale; MEPs, motor evoked potentials; MFT, manual function test; MMSE, mini-mental state examination; MRS, Modified Rankin Scale; NA, not available; NHPT, Nine Hole Peg Test; NIHSS, National Institutes of Health Stroke Scale; RCT, randomized clinical trial; SIS, Stroke impact Scale; TBS, theta burst stimulation; tDCS, transcranial direct current stimulation; 1TPDT_h_t, Two Point Discrimination Test; UE-FM, Upper Extremity Fugl-Meyer Motor Function; +, better results in the experimental group compared to the control group; –, worse results in the experimental group compared to the control group; = , no differences between experimental and control groups.

## Author contributions

GL and SP proposed the research idea, wrote the background and conclusion, and contributed to the literature review. AA supported the literature review and helped to revise the manuscript. SP prepared the manuscript for submission. All authors have read and approved the final version of the manuscript.
